# Endemic and Epidemic Lineages of *Escherichia coli* that Cause Urinary Tract Infections 

**DOI:** 10.3201/eid1410.080102

**Published:** 2008-10

**Authors:** Amee R. Manges, Helen Tabor, Patricia Tellis, Caroline Vincent, Pierre-Paul Tellier

**Affiliations:** McGill University, Montréal, Québec, Canada (A.R. Manges, P. Tellis, C. Vincent, P.-P. Tellier); National Microbiology Laboratory, Winnipeg, Manitoba, Canada (H. Tabor)

**Keywords:** Escherichia coli, molecular epidemiology, urinary tract infections, extraintestinal infections, antimicrobial drug resistance, research

## Abstract

Women with urinary tract infections (UTIs) in California, USA (1999–2001), were infected with closely related or indistinguishable strains of *Escherichia coli* (clonal groups), which suggests point source dissemination. We compared strains of UTI-causing *E. coli* in California with strains causing such infections in Montréal, Québec, Canada. Urine specimens from women with community-acquired UTIs in Montréal (2006) were cultured for *E. coli*. Isolates that caused 256 consecutive episodes of UTI were characterized by antimicrobial drug susceptibility profile, enterobacterial repetitive intergenic consensus 2 PCR, serotyping, *Xba*I and *Not*I pulsed-field gel electrophoresis, multilocus sequence typing, and phylogenetic typing. We confirmed the presence of drug-resistant, genetically related, and temporally clustered *E. coli* clonal groups that caused community-acquired UTIs in unrelated women in 2 locations and 2 different times. Two clonal groups were identified in both locations. Epidemic transmission followed by endemic transmission of UTI-causing clonal groups may explain these clusters of UTI cases.

## Abstract 2

These organisms caused community-acquired UTI-causing *Escherichia coli*

Community-acquired extraintestinal infections with *Escherichia coli* range in frequency from 6 to 8 million cases of uncomplicated cystitis per year to 127,500 cases of sepsis per year in the United States (*1*). Urinary tract infections (UTIs) caused by *E*. *coli* are one of the most common extraintestinal infections in women and, because of their high incidence, are the focus of most epidemiologic studies. The source of *E*. *coli* for these infections is a person’s intestinal tract; however, how these *E*. *coli* are acquired by the gut is unclear. Risk factors that lead to intestinal colonization with extraintestinal *E*. *coli* differ from factors associated with development of infection.

Young, otherwise healthy, sexually active women have the highest risk for community-acquired UTIs. The main risk factors for UTI are recent and frequent sexual intercourse, contraceptive use, and a history of UTIs (*2**,**3*). Treatment for UTIs usually involves a short course of an antimicrobial drug, such as trimethoprim-sulfamethoxazole (TMP-SMZ). Over the past decade, the prevalence of drug resistance in *E*. *coli* has increased dramatically, complicating management of these infections. Across the United States and Canada, urinary tract isolates of *E*. *coli* from outpatient clinics showed increased resistance to TMP-SMZ and ampicillin (*4*). A more serious concern has been the gradual increase in fluoroquinolone (e.g., ciprofloxacin) resistance among UTI isolates (*5*).

There is increasing evidence that the *E*. *coli* that cause UTIs and other extraintestinal infections may be responsible for community-wide epidemics. In 1986–1987, *E*. *coli* O15:K52:H1 caused an outbreak of community-acquired UTIs and septicemia in South London, England (*6*). The distinctive drug resistance profile of this clonal group contributed to its recognition in London and other areas of Europe and the United States (*7**,**8*). Other outbreaks of UTI caused by *E*. *coli* have been described and include a cluster of UTI cases in Copenhagen, Denmark, caused by *E*. *coli* O78:H10 and a larger outbreak in Calgary, Alberta, Canada, caused by extended-spectrum β-lactamase (ESBL)–producing *E*. *coli* (*9**,**10*).

In 2001, we reported that a multidrug-resistant *E*. *coli* clonal group designated clonal group A (CgA), defined by an enterobacterial repetitive intergenic consensus 2 (ERIC2) PCR and characterized by O11, O77, O17, and O73:K52:H18 serotypes, caused 11% of all *E*. *coli* UTIs and 49% of all TMP-SMZ–resistant *E*. *coli* UTIs in 1 California, USA, community over a 4-month period (*11*). Members of this clonal group were responsible for drug-resistant UTIs in university communities in Michigan and Minnesota and a community in Colorado (*12*), and for pyelonephritis in several states (*13*). We also identified additional clonal groups in a second cross-sectional study in Berkeley, California (*14*).

Identification of outbreak strains of *E*. *coli* that cause extraintestinal infections suggests that point sources, possibly contaminated food, may be responsible for local spread of genetically related *E*. *coli* strains in the community. Recent work in the United Kingdom has focused on a possible link between the increase in ESBL-producing *E*. *coli* and food animal production. An estimated 30,000 cases of human infection with ESBL-producing *E*. *coli* occur each year in the United Kingdom, and studies have found epidemic strains of ESBL-producing *E*. *coli* in the United Kingdom and throughout the world (*15*–*17*). The Health Protection Agency has suggested that imported chicken may be a route for introduction of ESBL-producing *E*. *coli* into the United Kingdom. Recent research by this agency did not identify a direct link between ESBL-positive strains of *E*. *coli* and chickens and humans (*18*), but other investigators found evidence for a link between drug resistance and specific genotypes of extratintestinal *E*. *coli* in animal food products and human infections in Minnesota and Washington, DC (*19*–*21*).

To further investigate the molecular epidemiology of disseminated *E*. *coli* clonal groups that cause UTIs, we conducted a cross-sectional study in a population of university women from Montréal, Québec, Canada, with UTI caused by *E*. *coli* and compared these organisms with those isolated from women with UTI in California. We sought to identify women in similar risk groups, but at different times and in different locations, to determine whether unrelated women with UTIs caused by indistinguishable strains of *E*. *coli* could be identified, and to determine whether the distribution was identical of clonal groups that were causing UTIs in these 2 communities.

## Methods

### Study Design

We conducted a cross-sectional study in collaboration with the Student Health Services at McGill University in Montréal in 2006. Eligible women 18–45 years of age who came to the health center with a suspected UTI were enrolled in the study. A UTI was clinically defined as >2 symptoms suggestive of this infection and included dysuria, increased urinary frequency or urgency, pyuria, hematuria, and >10^2^ CFUs of *E*. *coli*/mL of clean-catch urine. If a woman had >1 UTIs during the study period, only data concerning the first UTI was eligible for inclusion in the analyses. Details of studies in California have been reported (*11*,*14*). The study protocol was reviewed and approved by the McGill University, Institutional Review Board (A01-M04–05A).

### Isolation of *E. coli*

Urine samples were immediately cultured on Uricult (Orion Diagnostica, Espoo, Finland) MacConkey/cysteine lactose electrolyte–deficient agar dip slides. One arbitrarily selected colony (or multiple if morphologically different colonies were present) was selected from the MacConkey side. Lactose- and indole-positive colonies were presumptively identified as *E*. *coli* (*22*). Those isolates that were either lactose or indole negative were cultured on CHROMagar orientation plates (Becton Dickinson BBL Diagnostics, Sparks, MD, USA) and tested for lysine and ornithine decarboxylases (Moeller decarboxylase tests; PML Microbiologicals, Mississauga, Ontario, Canada). The reference strains used for carboxylase testing included *Klebsiella pneumoniae* (American Type Culture Collection [ATCC] no. 13883) and *Enterobacter cloacae* (ATCC no. 13047). Those isolates that were classified as *E*. *coli* on the CHROMagar plates and positive for lysine and ornithine decarboxylases were presumptively identified as *E*. *coli*. One *E*. *coli* isolate from each urine culture was arbitrarily selected for further analysis.

### Antimicrobial Drug Susceptibility

Isolates were screened for susceptibility to TMP-SMZ, ciprofloxacin, cephalothin, nitrofurantoin, ampicillin, chloramphenicol, streptomycin, and tetracycline by the disk diffusion assay (Becton Dickinson BBL Diagnostics). *E*. *coli* strain ATCC 25922 was used as the reference strain. Isolates were defined as resistant, intermediate, or susceptible to each antimicrobial drug according to Clinical and Laboratory Standards Institute interpretive criteria (*23*). Isolates with intermediate resistance were defined as susceptible.

### ERIC2 PCR Fingerprinting

All *E*. *coli* isolates were screened by using the ERIC2 PCR fingerprinting assay (*24*). Images of electrophoretic patterns were scanned into a software program (GelCompar II version 3.5; Applied Maths Inc., Austin, TX, USA) for analysis. Dendrograms based on ERIC2 PCR patterns were inferred from the Dice similarity coefficient matrix generated by GelCompar by the unweighted pair group method with arithmetic averages. Isolates with fingerprints that were indistinguishable on visual inspection or by GelCompar II version 3.5 (Applied Maths Inc.) analysis were grouped and selected for further typing.

### Pulsed-Field Gel Electrophoresis

*Xba*I and *Not*I pulsed-field gel electrophoresis (PFGE) was conducted on all putative clonal isolates, as defined by ERIC2 PCR (*25*). Isolates showing <6 band differences in their patterns were considered to be possibly related according to the criteria of Tenover et al. (*26*). Images of patterns were scanned into GelCompar II version 3.5 and analyzed as for ERIC2 PCR.

### Serotypes

Serotyping was performed for Montréal *E*. *coli* isolates that were indistinguishable by ERIC2 PCR. O and H serotyping was performed by the Enteric Diseases Program at the National Microbiology Laboratory, Winnipeg, Manitoba, Canada, by using established protocols. Isolates from California were evaluated for serogroup only at the *E*. *coli* Reference Center (Pennsylvania State University, University Park, PA, USA). Isolates that were motile but non reactive with O or H antiserum were classified as nontypeable (OUNTYPE) and those that were nonmotile were denoted (HNM).

### Multilocus Sequence Typing and Determination of Phylogenetic Group

Multilocus sequence typing (MLST) was performed as described (*27*). Gene amplification and sequencing were performed by using the primers specified at the *E*. *coli* MLST website (http://web.mpiib-berlin.mpg.de/mlst/dbs/Ecoli). Allelic profile and sequence type (ST) determinations were assigned according to the *E*. *coli* MLST website scheme. The major *E*. *coli* phylogenetic group (A, B1, B2, and D) was determined by using a multiplex PCR (*28*).

### Clonal Group

A clonal group was defined as >2 *E*. *coli* isolates showing indistinguishable patterns by ERIC2 PCR. These groups were given letter designations, such as CgA. Clonal group designations assigned for the California study isolates were retained (CgA to CgG), and clonal groups identified in Montréal were assigned new letter designation beginning with CgH. To support categorization of these clonal groups, isolates showing indistinguishable ERIC2 PCR patterns were also evaluated by PFGE, serotyping, drug susceptibility testing, MLST, and phylogenetic typing.

### Statistical Analyses

All analyses were conducted by using Stata version 9.0 (Stata Corporation, College Station, TX, USA). Proportions and 95% confidence intervals (CIs) were estimated. Differences in proportions were assessed by χ^2^ tests. Statistical significance was defined by p<0.05.

## Results

### Study Participants

From January 2006 to January 2007, 656 urine samples were submitted. *E*. *coli* was isolated from 300 urine samples obtained from 256 women in Montréal. Only samples from the first UTI were included in the analyses. A total of 311 (47%) samples yielded no bacteria, and 45 (7%) contained an organism other than *E*. *coli*. Results for the *E*. *coli* isolated from these 256 women with UTIs were compared with results for *E*. *coli* isolated from 434 women with UTIs in California (1999–2001).

### Antimicrobial Drug Susceptibility

Antimicrobial drug resistance for the Montréal and California isolates is summarized in Table 1. For the drugs tested, isolates from Montréal showed comparable resistance levels to those from California, although resistance to TMP-SMZ was higher in isolates from California (20% in California vs. 14% in Montréal; p = 0.07) and ciprofloxacin resistance was slightly higher in isolates from Montréal (2% in California vs. 4% in Montréal; p = 0.06). Resistance to nitrofurantoin was not detected in isolates from either location.

**Table 1:** Antimicrobial drug resistance of Escherichia coli*

Characteristic	Berkeley, California, USA†	Montréal, Québec, Canada‡	p value§
Total primary *E. coli*	434	256	
Drug	No. (%) resistant		
Trimethoprim- sulfamethoxazole	85 (20)	36 (14)	0.07
Cephalothin	11 (3)	7 (3)	0.90
Ciprofloxacin	8 (2)	11 (4)	0.06
Nitrofurantoin	0	0	
Ampicillin	ND	83 (32)	ND
Tetracycline	ND	40 (16)	ND
Chloramphenicol	ND	7 (3)	ND
Streptomycin	ND	48 (19)	ND

*ND, not done. †October 1999–January 2000 and October 2000– January 2001. ‡January 2006–January 2007 §By χ^2^ test.

### ERIC2 PCR Fingerprinting

ERIC2 PCR fingerprinting identified 4 clonal groups (CgA, CgC, CgH, and CgI) among Montréal isolates (data not shown). The prevalence of these clonal groups in Montréal in 2006 was 13 CgA (5%, 95% CI 0.03–0.09), 10 CgC (4%, 95% CI 0.02–0.07), 7 CgI (3%, 95% CI 0.01–0.06), and 5 CgH (2%, 95% CI 0.01–0.04). CgA and CgC were identified from both study sites. In the California studies, 32 CgA isolates (7%, 95% CI 0.05–0.10) and 12 CgC isolates (3%, 95% CI 0.01–0.05) were identified. Clonal groupings were confirmed by PCR reamplification, and these groupings also included representatives of clonal groups identified in the California studies (*11*,*14*).

CgH was uniformly resistant to ampicillin and streptomycin and susceptible to all other drugs tested. CgC was susceptible to all drugs tested (except for 1 isolate that was resistant to ampicillin). CgA was primarily resistant to TMP-SMZ and ampicillin; resistance to the other drugs varied. CgI showed the most extensive resistance. This group was resistant to ciprofloxacin and TMP-SMZ, and 2 members of CgI were resistant to 5 drugs. Drug-resistance profiles for each clonal group member from both study sites are shown in Table 2 and the Appendix Table.

**Table 2:** Characteristics of clonal isolates of *Escherichia coli* from women with urinary tract infections, Montréal, Québec, Canada, 2006

Isolate no.	Genotype*	Serotype	MLST†	Phy‡	Date of infection	Antimicrobial drug resistance profile§							
						CIP	CEP	NIT	TMP-SMZ	AMP	CAM	STR	TET
362	C	O1:H7	ST95	B2	2006 Jan 23	0	0	0	0	0	0	0	0
363	C	O1:H7			2006 Jan 23	0	0	0	0	0	0	0	0
413	C	O18:H7	ST95	B2	2006 Feb 13	0	0	0	0	0	0	0	0
414	C	O1:H7			2006 Feb 13	0	0	0	0	0	0	0	0
439	C	O1:H7			2006 Feb 28	0	0	0	0	0	0	0	0
762	C	O1:K1:H7	ST95	B2	2006 Sep 28	0	0	0	0	0	0	0	0
767	C	O1:K1:H7			2006 Sep 29	0	0	0	0	1	0	0	0
782	C	O2:K1:H7	ST95	B2	2006 Oct 10	0	0	0	0	0	0	0	0
957	C	O1:H7			2007 Jan 1	0	0	0	0	0	0	0	0
958	C	O1:H7	ST95	B2	2007 Jan 5	0	0	0	0	0	0	0	0
412	H	O6:H1	ST73	B2	2006 Feb 13	0	0	0	0	1	0	1	0
415	H	O6:H1			2006 Feb 13	0	0	0	0	1	0	1	0
422	H	O6:H1			2006 Feb 16	0	0	0	0	1	0	1	0
459	H	O6:H1			2006 Mar 10	0	0	0	0	1	0	1	0
471	H	O6:H1			2006 Mar 16	0	0	0	0	1	0	1	0
385	A	OR:H18	ST69	D	2006 Jan 30	0	0	0	0	1	0	1	0
434	A	O73:H18	ST69	D	2006 Feb 27	0	0	0	0	0	0	0	0
498	A	O77/17:H18	ST69	D	2006 Mar 24	0	0	0	1	1	0	1	0
713	A	OUNTYPE: HNM	ST69	D	2006 Sep 11	0	0	0	1	1	0	1	0
724	A	O15:H18	ST69	D	2006 Sep 13	0	0	0	0	0	0	0	0
799	A	OUNTYPE: H18	ST69	D	2006 Oct 16	0	0	0	1	1	0	1	0
839	A	O17:H18	ST69	D	2006 Nov 2	0	0	0	1	0	0	0	0
860	A	O25:H18	ST69	D	2006 Nov 11	0	0	0	0	0	1	0	1
868	A	OUNTYPE: H18	ST69	D	2006 Nov 15	0	0	0	0	0	0	0	0
908	A	O17:H18	ST69	D	2006 Nov 30	0	0	0	1	1	0	0	1
912	A	O17:H18	ST69	D	2006 Nov 30	0	0	0	1	1	0	1	0
913	A	O17:HNM	ST69	D	2006 Dec 1	0	0	0	0	0	0	0	1
956	A	OUNTYPE: H18	ST69	D	2007 Jan 3	0	0	0	0	0	1	1	1
375	I	O25:H4	ST131	B2	2006 Jan 25	1	0	0	0	1	0	0	0
452	I	O25:H4	ST131	B2	2006 Mar 8	1	0	0	1	1	0	0	1
544	I	O25:H4			2006 Apr 19	1	0	0	0	0	0	0	0
550	I	O25:HNM	ST131	B2	2006 Apr 20	1	0	0	1	1	0	1	1
760	I	O25:H4			2006 Sep 28	1	0	0	1	1	0	0	1
783	I	O25:H4			2006 Oct 11	1	0	0	0	1	0	0	0
841	I	O25:H4	ST131	B2	2006 Nov 3	1	0	0	1	1	0	1	1

*Determined by ERIC2 PCR (*24*). †MLST, multilocus sequence typing, according to Tartof et al. (*27*); ST, sequence type. ‡Phy, phylogenetic group, determined by multiplex PCR (*28*). §0, sensitive; 1, resistant, according to Clinical and Laboratory Standards Institute interpretative criteria (*23*). CIP, ciprofloxacin; CEP, cephalothin; NIT, nitrofurantoin; TMP-SMZ, trimethoprim-sulfamethoxazole; AMP, ampicillin; CAM, chloramphenicol; STR, streptomycin; TET, tetracycline.

### Pulsed-Field Gel Electrophoresis

**Figure 1:**
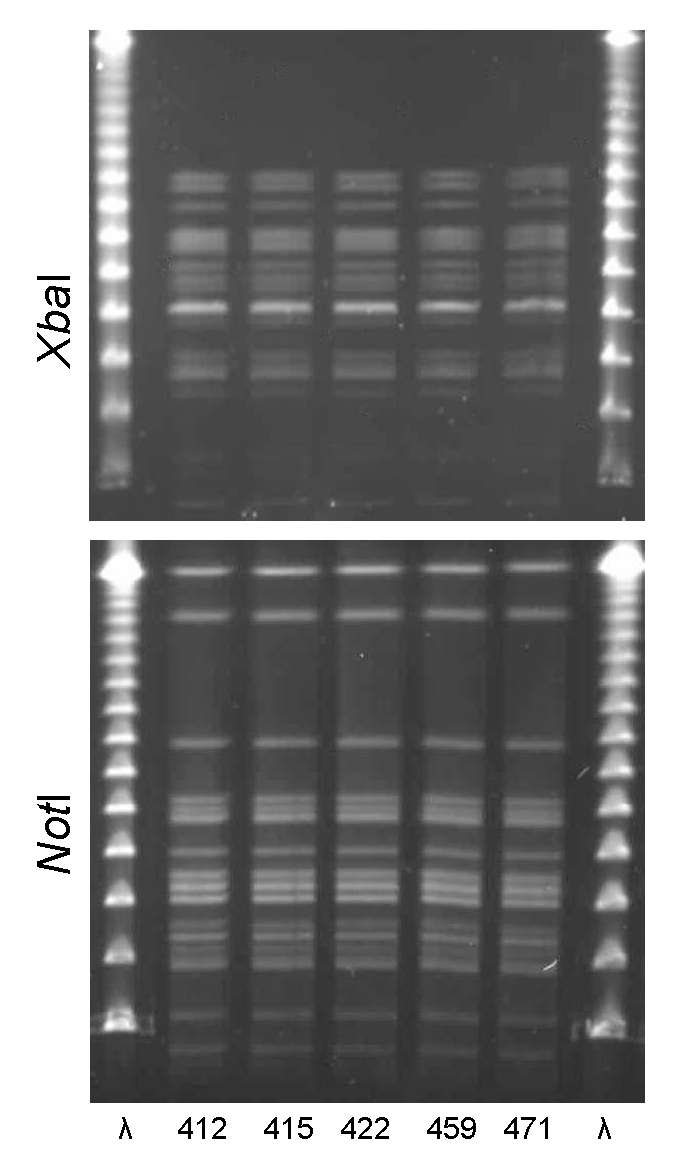
*Xba*I and *Not*I pulsed-field gel electrophoresis patterns for clonal group H *Escherichia coli* isolated from women with urinary tract infections in Montréal, Québec, Canada, 2006. The 5 isolates shown were serogroup O6:H1. First and last lanes, bacteriophage λ.

**Figure 2:**
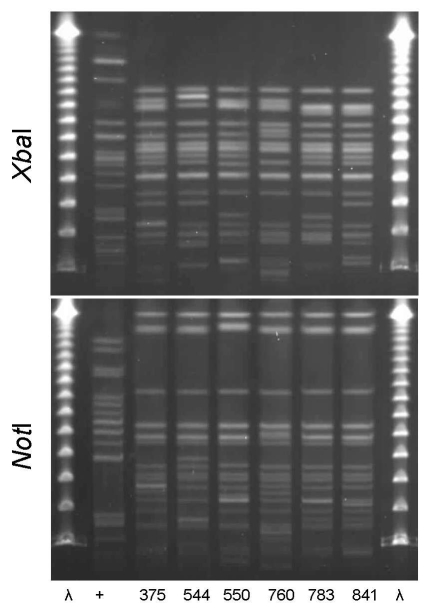
*Xba*I and *Not*I pulsed-field gel electrophoresis patterns for clonal group I *Escherichia coli* isolated from women with urinary tract infections in Montréal, Québec, Canada, 2006. The 6 isolates shown were resistant to ciprofloxacin and in serogroup O25:H4. First and last lanes, bacteriophage λ; lane +, positive control.

**Figure 3:**
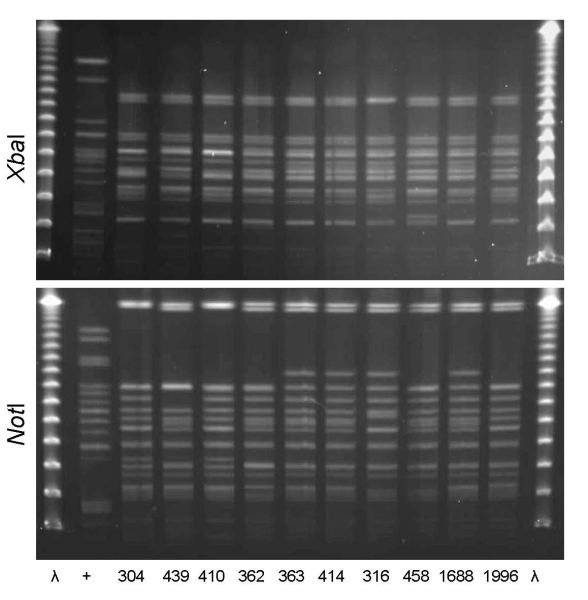
*Xba*I and *Not*I pulsed-field gel electrophoresis patterns for clonal group C *Escherichia coli* isolated from women with urinary tract infections in Montréal, Québec, Canada, 2006 (lanes 304, 439, 362, 363, and 414) and Berkeley, California, USA, 1999–2001 (lanes 410, 316, 458, 1688, and 1996). The 10 isolates shown were susceptible to all antimicrobial drugs tested and included serogroups O1, O2, or O18. First and last lanes, bacteriophage λ; lane +, positive control.

**Figure 4:**
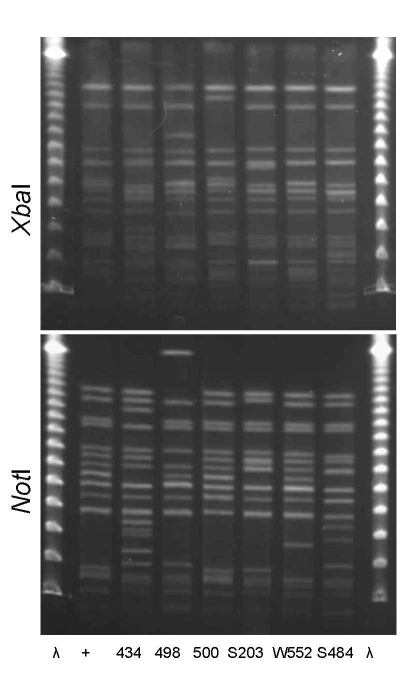
*Xba*I and *Not*I pulsed-field gel electrophoresis patterns for clonal group A *Escherichia coli* isolated from women with urinary tract infections in Montréal, Québec, Canada, 2006 (lanes 434 and 498) and Berkeley, California, USA, 1999–2001 (lanes 500, S203, W552, and S484). Antimicrobial drug resistance phenotypes and serogroups (O11, O17, O77, and O73) varied within and between the 2 study locations. First and last lanes, bacteriophage λ; lane +, positive control.

PFGE confirmed the presence of 4 clonal groups among the Montréal isolates. CgH was found only in Montréal and showed indistinguishable *Xba*I and *Not*I PFGE patterns (Figure 1). CgI was also found only in Montréal and could be considered possibly related by the criteria of Tenover et al. (*26*) (Figure 2). Patterns of CgC isolates (Figure 3) identified in California and Montréal differed by <6 bands, regardless of restriction enzyme used. The PFGE results for CgA varied the most among all clonal groups from Montréal; in some cases, the PFGE patterns showed >6 band differences (Figure 4).

### Serotypes

Serotype results for all clonal *E*. *coli* isolates identified in California and Montréal are shown in Table 2 and the Appendix Table. Serotyping was consistent within each clonal group, except for CgA, which showed 6 serogroups (O11, O77, O17, O73, O25, and O15) although O25 and O15 occurred only once. The complete serotype for CgA was O11/O17/O77/O73:K52:H18. CgC from both study locations showed the same serotype (O1/O18/O2:K1:H7).

### MLST

Sequence types for selected members of each clonal group from the California and Montréal studies were determined (Table 2 and Appendix Table). All sequence types were internally consistent within the clonal group. CgC and CgA isolates from both study sites showed the same sequence types (ST95 and ST69, respectively). CgH, CgB, and CgD showed the same sequence type (ST73). These 3 clonal groups also showed similar serogroups and phylogenetic groups but showed variable ERIC2 PCR and PFGE patterns; thus, they were not placed in the same clonal grouping.

### Phylogenetic Group

Phylogenetic group was determined for selected members of each clonal group (Table 2; Appendix Table). All phylogenetic group assignments were internally consistent within the clonal group and classified as either phylogenetic group B2 or D; both are typically associated with extraintestinal *E*. *coli*.

### Time Cluster Analyses

**Figure 5:**
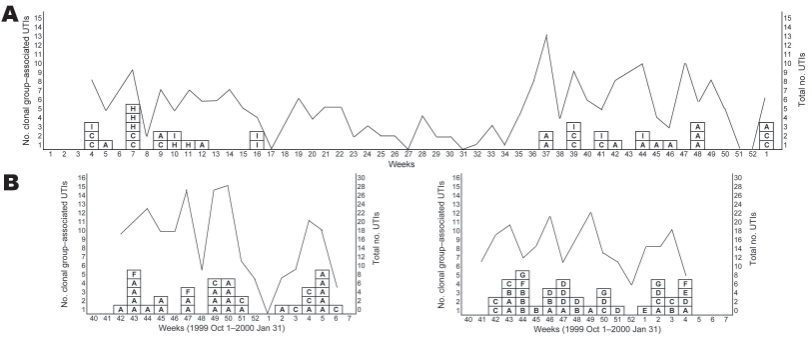
Temporal patterns of cases of urinary tract infections (UTIs) with *Escherichia coli* clonal groups by week in Montréal, Québec, Canada, 2006 (A), and Berkeley, California, USA, 1999–2001 (B). Clonal groups are identified by letters in boxes. Lines indicate the total number of UTIs with *E*. *coli* in each week for each study site. Samples were not analyzed during February–October 2000 in Berkeley.

In considering the hypothesis of endemic versus epidemic transmission of these clonal groups, temporal clustering is a useful factor. Figure 5 shows the temporal pattern by week of UTI cases for all clonal groups in Montréal (Figure 5, panel A) and in California (Figure 5, panel B). Fluctuation in the number of *E*. *coli* UTIs over time corresponds closely to observation of clonal group–associated UTI cases. These results show clustering of some clonal groups, e.g., 3 of the 5 UTIs caused by CgH occurred in Montréal during week 7, and CgH did not appear again in Montréal after week 11. In California, CgA was present more frequently between October 1999 and February 2000 and dropped by 39% between the 2 sampling periods (*14*). CgB and CgD occurred exclusively in the second phase of the California study (Figure 5, panel B). Other clonal groups appeared throughout the year, although they often clustered by week. CgC was present during both data collection periods in California and caused UTIs throughout 2006 in Montréal. No clonal group members were identified during the summer in Montréal. However, this period corresponded to a decrease in the number of UTI cases at the student health services because of lower summer university enrollment (see total *E*. *coli* UTI by week, Figure 5).

## Discussion

This study confirms the presence of drug-resistant, genetically related, and, in some cases, temporally clustered *E*. *coli* clonal groups (CgH, CgI, CgC, and CgA) that caused community-acquired UTIs in unrelated women in 2 locations and at different times. Drug resistance did not differ considerably between the 2 study sites, nor did the overall percentage of UTI caused by clonal groups: 4% (95% CI 0.10–0.18) in Montréal and 16% (95% CI 0.13–0.20) in California. Two clonal groups (CgA and CgC) were identified in both study locations, indicating widespread dissemination. These clonal groups shared common serogroups, PFGE patterns, drug-susceptibility profiles, MLST patterns, and phylogenetic groups. CgA isolates identified in Montréal did not show the same degree of genetic homogeneity as CgA isolates identified in the original California studies (*11**,**14*). CgA has also been recognized in many other locations and may represent a lineage that has been spreading over a longer period than other more genetically homogenous clonal groups identified (*6**,**11**,**14**,**29*). CgC members isolated from both study locations showed similar PFGE patterns, as well as common serotypes, MLST patterns, and phylogenetic groups, which suggest that these isolates are likely related. The fully susceptible nature of the CgC group and the similar drug resistance levels at the 2 study sites suggest that drug resistance or pressure may not have contributed to its selection and dissemination.

Of the 4 clonal groups, 3 showed resistance to >1 antimicrobial drugs. Most worrisome was CgI, which was resistant to ciprofloxacin and TMP-SMZ, drugs commonly used to treat patients with UTIs. Two members of CgI were resistant to 5 drugs. Identification of CgI serotype O25:H4 is also important because this serogroup and its drug resistance profile have been identified in a recent report on an emerging CTX-M type ESBL-producing *E*. *coli* (serotype O25:H4 and ST131) found worldwide (*30*). A possible link between the O25:H4 *E. coli* clonal group identified in Montréal and this emerging ESBL-producing *E*. *coli* clonal group should be investigated.

Temporal clustering (Figure 5) of these clonal groups from the 2 study sites was observed. Clonal groups tended to be identified in women on the same day and week or in adjacent weeks; CgH in Montréal and CgA in California followed this pattern. However, many of the clusters caused by these clonal groups did occur sporadically across the entire study period. The observed correlation between increased incidence of total *E*. *coli* UTIs and increased incidence of clonal group–associated UTIs may be a function of having sufficient numbers of UTIs to be able to detect these clonal groups of *E*. *coli*. However, underlying fluctuations in community-wide dynamics of these *E*. *coli* clonal groups (in a human or environmental reservoir) may influence the overall number of clinical infections.

One strength of our study is the ability to directly estimate the proportion of UTIs caused by each clonal group in the study communities. Because the study included all consecutive UTI specimens from a defined population and all *E*. *coli* were cultured and analyzed, it was possible to produce unbiased estimates of these proportions. Laboratory-based studies may overestimate prevalence of drug resistance, which in turn may bias the estimated proportion of clonal groups detected when specimens from recurrent, relapse, or complicated UTIs are disproportionately represented in the study samples.

One limitation of our study is the lack of epidemiologic data on possible *E*. *coli* transmission routes. Lack of epidemiologic information makes it impossible to determine what specific risk factors led groups of women to become infected with indistinguishable strains of *E*. *coli*. Therefore, detection of a specific transmission route (e.g., foodborne) could not be directly addressed in this study. However, an earlier study, on the basis of epidemiologic data, has implicated frequent consumption of chicken and pork in the development of drug-resistant UTIs (*31*). Also, limited reproducibility of the ERIC2 PCR may have contributed to an underestimation of the number of clonal groups, particularly those clonal groups with only a few members (*32*). However, additional genotypic and phenotypic analyses applied to these isolates contributed to the valid classification of these clonal groups.

Genetic homogeneity of the clonal groups identified in this study (CgH, CgC, and CgI), in addition to similar observations from other reports (*6**,**10**,**17*), suggests that these clonal groups are circulating in humans, most probably as part of the intestinal reservoir, and that they contribute to a sizable fraction of UTIs in the community. However, the degree of relatedness within each clonal group varied. For example, certain clonal groups (notably CgH) were highly clustered in time and showed indistinguishable genetic and other characteristics, which suggests local and recent transmission. Other clonal groups showed more diversity (e.g., CgA), possibly reflecting long-term, endemic transmission.

These results suggest 3 competing or coincident questions. First, do local, punctuated epidemics of specific strains or clonal groups occur as observed in this and earlier studies (*6**,**10**,**33*)? Second, do these clonal groups belong to a set of fairly conserved endemic clonal groups that are adapted for persistent and predominant colonization of the intestinal tract, and which have spread widely in human communities over varying periods of time (*6**,**10**–**14**,**29**,**33**,**34*)? Third, a combination of the first and second questions, are there periodic (epidemic) introductions of *E*. *coli* clonal groups in a community by an external source followed by endemic transmission? Already some evidence has indicated that animal-based foods or retail meats may contribute to the spread of these clonal groups (*19*–*21*). The number of infections, timing, and diverse locations in which these clonal groups are found argues against the possibility that person-to-person or household transmission contributes to our findings. However, limited local spread by these routes by certain clonal groups cannot be ruled out (*35**–**38*).

Positive and negative implications are associated with our results. One positive implication is that identification of lineages or clonal groups of *E*. *coli* that cause a sizeable fraction of community-acquired UTIs or extraintestinal infections may contribute to rational development of therapies and prevention strategies targeted toward these lineages. One negative implication is that tracing transmission routes and understanding the dynamics of these *E*. *coli* in external reservoirs and in human populations will be difficult and may impede possible control efforts, although ongoing attempts are under way to screen retail meats as a potential reservoir.

Annual incidence of UTIs and other community-acquired extraintestinal infections is high, in the millions, worldwide. Although each clonal group may account for a small fraction of all UTIs in a community, the high incidence of these infections implies that these clonal groups may contribute substantially to the overall extent of extraintestinal infections caused by *E. coli*. Furthermore, these clonal groups contribute, not only to uncomplicated infections such as cystitis, but also to severe infections such as pyelonephritis and septicemia (*13**,**39**,**40*). At a minimum, 10%–20% of these infections may be caused by 1 of a small set of extraintestinal pathogenic *E*. *coli* clonal groups, which are commonly resistant to >1 drugs. These facts point to the public health importance of understanding these *E*. *coli* lineages and their dynamics in the community and possible environmental reservoirs.

## Supplementary Material

Appendix Table Characteristics of clonal isolates of Escherichia coli from women with urinary tract infections, Berkeley, California, USA, 1999-2001
