# Supplementary material for: Endemic and Epidemic Lineages of Escherichia coli that Cause Urinary Tract Infections
Source: Emerg Infect Dis. 2008 Oct;14(10):1575–83. doi: 10.3201/eid1410.080102 (PMC2609861; doi:10.3201/eid1410.080102)
Supplement: Appendix Table — Characteristics of clonal isolates of Escherichia coli from women with urinary tract infections, Berkeley, California, USA, 1999-2001 [file 08-0102_appT-s1.pdf]

Appendix Table. Characteristics of clonal isolates of *Escherichia coli* from women with urinary tract infections, Berkeley, California, USA, 1999–2001

ID no.	Genotype*	O serogroup	MLST†	Phy‡	Date of infection	Antimicrobial drug resistance profile§			
						CIP	CEP	NIT	TMP-SMZ
3	A				1999 Oct 11	0	0	0	1
34	A	O17/O77			1999 Oct 18	0	0	0	0
38	A	O11			1999 Oct 18	0	0	0	1
44	A	O77	ST69		1999 Oct 19	0	0	0	1
46	A	O11			1999 Oct 19	0	0	0	1
102	A	O11	ST69	D	1999 Oct 29	0	0	0	1
120	A				1999 Nov 3	0	0	0	1
135	A				1999 Nov 4	0	0	0	1
203	A	O11			1999 Nov 16	0	0	0	1
220	A	O77	ST69		1999 Nov 17	0	0	0	1
264	A				1999 Nov 29	0	0	0	1
283	A	O77	ST69		1999 Nov 30	0	0	0	1
294	A	O11			1999 Dec 1	0	0	0	1
323	A	O11			1999 Dec 6	0	0	0	1
325	A				1999 Dec 6	0	0	0	0
354	A				1999 Dec 9	0	0	0	1
363	A				1999 Dec 11	0	0	0	1
383	A	O11			1999 Dec 16	0	0	0	1
403	A	O11	ST69		2000 Jan 7	0	0	0	1
431	A	O11	ST69		2000 Jan 18	0	0	0	1
470	A	O77			2000 Jan 25	0	0	0	1
476	A				2000 Jan 25	0	0	0	1
477	A	O11	ST69		2000 Jan 25	0	0	0	1

486	A	O11			2000 Jan 27	0	0	0	1
490	A	O11			2000 Jan 27	0	0	0	1
1663	A	Mixed			2000 Oct 16	0	0	0	1
1683	A				2000 Oct 19	0	0	0	0
1757	A				2000 Nov 6	0	0	0	0
1792	A	O11			2000 Nov 14	0	0	0	1
1868	A				2000 Dec 1	0	0	0	0
2013	A	O11			2001 Jan 12	0	0	0	1
2058	A	O11			2001 Jan 22	0	0	0	1
1664	B	O2			2000 Oct 17	0	0	0	0
1697	B	O6	ST73	B2	2000 Oct 23	0	0	0	0
1699	B	O6			2000 Oct 23	0	0	0	0
1715	B	O6	ST73	B2	2000 Oct 25	0	0	0	0
1756	B	O2			2000 Nov 4	0	0	0	0
1768	B	O6	ST73	B2	2000 Nov 8	0	0	0	0
1814	B	O2	ST73	B2	2000 Nov 20	0	0	0	0
2042	B	O6	ST73	B2	2001 Jan 19	0	0	0	0
316	C	O1	ST95	B2	1999 Dec 3	0	0	0	0
374	C	O18	ST95	B2	1999 Dec 13	0	0	0	0
410	C	O1			2000 Jan 11	0	0	0	0
448	C	O18			2000 Jan 19	0	0	0	0
458	C	O1	ST95	B2	2000 Jan 21	0	0	0	0
500	C	O1			2000 Jan 31	0	0	0	0
1642	C	O1			2000 Oct 12	0	0	0	0
1643	C	O18			2000 Oct 12	0	0	0	0
1688	C	O1	ST95	B2	2000 Oct 20	0	0	0	0
1897	C	O2			2000 Dec 5	0	0	0	0
1996	C	O1	ST95	B2	2001 Jan 9	0	0	0	0
2018	C	O1			2001 Jan 16	0	0	0	0

1758	D	O6	ST73	B2	2000 Nov 6	0	0	0	0
1789	D	O6			2000 Nov 14	0	1	0	1
1790	D	O6	ST73	B2	2000 Nov 14	0	0	0	1
1793	D	O83			2000 Nov 14	0	0	0	0
1827	D	O6			2000 Nov 22	0	0	0	0
1900	D	O6			2000 Dec 5	0	0	0	0
1923	D	O6	ST73	B2	2000 Dec 11	0	0	0	0
1994	D	O6			2001 Jan 8	0	0	0	0
2056	D	O6	ST73	B2	2001 Jan 22	0	0	0	1
1979	E	O82	ST420	D	2001 Jan 3	0	0	0	0
2062	E	O82			2001 Jan 22	0	0	0	0
61	F	O4:5W	ST12 complex	B2	1999 Oct 23	0	0	0	1
228	F	O4			1999 Nov 18	0	1	2	1
1694	F	Mixed			2000 Oct 23	0	0	0	1
2077	F	O4	ST12 complex	B2	2001 Jan 25	0	0	0	1
1702	G	O102,130			2000 Oct 24	1	1	0	1
1893	G	O102,130			2000 Dec 5	1	1	0	1
1991	G	O102,130	ST405	D	2001 Jan 8	1	1	0	1

*Determined by pulsed-field gel electrophoresis.

†MLST, multilocus sequence typing, according to Tartof et al. (27). Sequence types clonal Group A were also reported in this reference.

‡Phy, phylogenetic group, determined by multiplex PCR (28). Clonal group F isolates 61 (no ST match) and 2077 (ST493) belong to the sequence type 12 complex. Clonal group A isolates have been confirmed to belong to phylogenetic group D; therefore, only 1 representative clonal group A isolate was subjected to phylogenetic testing.

§0, sensitive; 1, resistant, according to Clinical and Laboratory Standards Institute interpretative criteria (23). CIP, ciprofloxacin; CEP, cephalothin; NIT, nitrofurantoin; TMP-SMZ, trimethoprim-sulfamethoxazole.
